# Tribological Performance of SPS-Fabricated Cu-HEA/Gr Composites Under Current-Carrying Sliding Contact

**DOI:** 10.3390/ma19173798

**Published:** 2026-09-07

**Authors:** Serdar Ozkaya, Müslim Çelebi, Harun Yanar, Abdullah Hasan Karabacak, Ertuğrul Çelik, Onur Güler, Abdulkadir Coskun, Dursun Murat Sekban

**Affiliations:** 1 Department of Metallurgical and Materials Engineering, Karadeniz Technical University, Trabzon 61080, Türkiye; muslimcelebi@ktu.edu.tr (M.Ç.); hasankarabacak@ktu.edu.tr (A.H.K.); onurguler@ktu.edu.tr (O.G.); 2 Department of Mechanical Engineering, Karadeniz Technical University, Trabzon 61080, Türkiye; 3 Trabzon Teknokent, UTS Engineering Services Industry Trade Limited Company, Trabzon 61080, Türkiye; 4 Department of Mechanical Engineering, Munzur University, Tunceli 62000, Türkiye; 5 Department of Marine Engineering Operations, Karadeniz Technical University, Trabzon 61080, Türkiye; abdulkadircoskun@ktu.edu.tr (A.C.);; 6 Trabzon Teknokent, WMS Engineering Services Industry Trade Limited Company, Trabzon 61080, Türkiye

**Keywords:** current-carrying sliding contact, high-entropy alloys, graphene, spark plasma sintering, Cu-based composites, electrical conductivity, tribology

## Abstract

Cu-based hybrid composites reinforced with AlCrFeCuNi high-entropy alloy (HEA) particles and graphene were successfully fabricated by spark plasma sintering (SPS) to investigate the combined effects of microstructure, mechanical properties, and tribological performance under electrical current. Microstructural observations revealed a dense and homogeneous distribution of HEA particles within the Cu matrix, while the addition of 2.0 wt.% graphene promoted noticeable grain refinement due to its grain boundary pinning effect. Although graphene addition provided beneficial effects in terms of grain refinement and electrical conductivity, it also increased the porosity from 2.49% to 5.08%. Consequently, the beneficial contribution of graphene to mechanical strengthening was substantially offset by the adverse effect of increased porosity, resulting in only a marginal increase in hardness from 127 HB to 129 HB. The addition of 2.0 wt.% graphene led to a pronounced change in the tribological behavior of the composites. The Cu–HEA exhibited relatively stable friction behavior but experienced a higher wear rate under all test conditions. In comparison, the Cu–HEA–2.0 Gr composite demonstrated a lower coefficient of friction and wear rate at 0–10 A, which is consistent with the possible formation of a graphene-rich lubricating surface layer. A clear transition occurred at 30 A, where the coefficient of friction and wear rate began to increase, while at 50 A, further increases in both parameters were observed, suggesting progressive disruption of the proposed protective surface layer under the investigated current-carrying sliding conditions.

## 1. Introduction

Current-carrying sliding contacts are essential components in many electromechanical systems, including slip rings, electrical brushes, rail transit systems, automotive electrical units, aerospace devices, and industrial equipment, where simultaneous mechanical motion and electrical transmission occur at the contact interface. Depending on the application and operating conditions, these systems may be exposed to high current levels reaching several tens of amperes or to high current densities concentrated over limited contact areas, making the selection of contact materials critically important [1,2,3]. Under such conditions, the contact interface is subjected not only to friction and mechanical wear, but also to Joule heating, arc-induced surface damage, oxidation, and thermally accelerated degradation, which may destabilize current transfer and eventually lead to severe surface deterioration and premature component failure [1,4]. Therefore, materials used in sliding electrical contact applications must simultaneously provide high electrical conductivity, efficient heat dissipation, mechanical integrity, wear resistance, and arc erosion resistance in order to ensure reliable long-term service performance [5].

Under such service conditions, the tribological behavior of current-carrying contact materials becomes considerably more complex than that observed under conventional dry sliding conditions [6]. In addition to contact pressure and frictional heating, the sliding interface is directly influenced by electrical current passage, which may induce local flash temperatures, arc-triggered surface damage, oxide film formation, softening, delamination, and electrically assisted material transfer [7,8]. As a result, the friction and wear response of electrical contact materials cannot be evaluated solely in terms of mechanical abrasion or adhesion, but must be interpreted through the coupled effects of mechanical, thermal, and electrical interactions [9]. In this regard, the tribo-electrical performance of a material is strongly governed by its hardness, microstructural stability, electrical and thermal conductivity, interfacial bonding, and the characteristics of the tribofilm developed during sliding. Consequently, advanced sliding contact materials are expected to satisfy multiple requirements simultaneously, including high electrical conductivity, efficient heat dissipation, adequate mechanical strength, low friction, high wear resistance, and stable contact behavior under current flow [7,10,11].

Accordingly, the development of appropriate materials for electrical contact systems has become a key issue in improving the reliability, durability, and service stability of current-carrying components used in various electromechanical applications [12]. Cu matrix composites are widely considered promising materials for such applications due to their excellent electrical and thermal conductivity, which are essential for stable current transfer and rapid heat dissipation [13,14]. Moreover, by introducing suitable reinforcing phases, the tribological performance, mechanical strength, and resistance to arc- and wear-induced surface damage of the Cu matrix can be markedly enhanced [15].

To address these requirements, extensive research has been devoted to Cu matrix composites reinforced with various lubricating and ceramic phases for improving both load-bearing capability and wear performance [16]. High-entropy alloys (HEAs) have recently emerged as highly promising candidates due to their unique compositional design and outstanding combination of mechanical and functional properties. Owing to severe lattice distortion, sluggish diffusion behavior, and synergistic elemental interactions, HEAs can exhibit high hardness, excellent thermal stability, superior resistance to wear, and good structural integrity under harsh service conditions [17,18]. These features make them particularly attractive as reinforcing phases for Cu-based composites intended for tribological applications. The AlCrFeCuNi system is especially noteworthy, as it combines the strengthening contribution of multiple metallic elements with good resistance to deformation and surface damage. Al may contribute to strengthening and phase stability, whereas Cr and Fe are well known for improving hardness and oxidation resistance. Ni can enhance structural robustness, and Cu-containing HEAs may provide a certain level of chemical and interfacial compatibility with a Cu matrix [19,20]. When introduced into copper as a reinforcement phase, AlCrFeCuNi particles can act as hard load-supporting constituents that reduce localized plastic deformation, limit the penetration of counterface asperities, and suppress severe plowing and adhesive wear. From a tribo-mechanical standpoint, such hard phases may also improve stress distribution at the sliding interface and enhance the microstructural stability of the composite under current-assisted friction conditions [7,21]. In parallel with HEAs, graphene has become one of the most attractive carbon-based reinforcements for Cu matrix composites due to its exceptional mechanical, electrical, and thermal properties. Graphene possesses extremely high intrinsic strength and elastic modulus, while also providing remarkable electrical conductivity and thermal transport capability. These characteristics make graphene particularly suitable for current-carrying tribological systems, where both conductive continuity and mechanical reinforcement are required [6,22]. In addition, graphene can function as a solid lubricant by forming a low-shear interfacial layer or transfer film during sliding. Such a tribofilm can reduce direct metal-to-metal contact, lower friction coefficient, minimize adhesive damage, and improve wear stability. In this way, graphene serves not only as a mechanical reinforcement, but also as a functional phase capable of improving interfacial sliding behavior and potentially stabilizing electrical contact conditions [23,24,25].

The successful fabrication of Cu-HEA/Gr MMCs, however, depends strongly on the processing route, since the final properties are highly sensitive to density, particle bonding, reinforcement dispersion, and microstructural integrity. Conventional sintering methods often involve long dwell times and high temperatures, which may promote grain coarsening, oxidation, undesirable interfacial reactions, or structural degradation of graphene [26,27]. For this reason, spark plasma sintering (SPS) has become an attractive processing method for advanced MMC systems. Owing to its rapid heating rate, short sintering duration, and simultaneous application of pressure, SPS enables efficient densification at relatively lower temperatures while minimizing grain growth and preserving reinforcement effectiveness [28,29]. In hybrid Cu-based composites, SPS can improve particle rearrangement, reduce porosity, strengthen matrix–reinforcement interfaces, and promote a refined and dense microstructure. These advantages are particularly important for sliding electrical contacts, since porosity, weak interfacial bonding, and heterogeneous phase distribution may directly impair both mechanical durability and electrical contact stability. Although Cu-based composites reinforced with ceramic particles, solid lubricants, and carbonaceous phases have been extensively studied, research on SPS-fabricated Cu-based MMCs simultaneously reinforced with AlCrFeCuNi HEA and graphene remains quite limited, especially under current-carrying sliding conditions. In particular, the relationship between microstructural evolution, hardness, and tribo-electrical performance in such hybrid composites has not yet been fully clarified. Recent studies have further demonstrated the importance of surface degradation and wear mechanisms in advanced metallic systems. However, the tribological response of SPS-fabricated Cu-based composites simultaneously containing AlCrFeCuNi HEA reinforcement and graphene under current-carrying sliding conditions remains insufficiently understood [29,30]. Likewise, the synergistic influence of a hard HEA phase and a lubricating graphene phase on friction, wear, and electrical contact-related behavior still requires systematic investigation. In our previous study, the influence of graphene content on the microstructural, mechanical, and dry-sliding tribological behavior of SPS-fabricated Cu–AlCrFeCuNi composites was systematically investigated [31], and the Cu–HEA–2.0 Gr composite exhibited the best overall tribological performance owing to the formation of a stable graphene-derived lubricating tribofilm. Nevertheless, Ref. [31] focused exclusively on conventional dry-sliding conditions and did not investigate the tribological response of the optimized Cu–HEA–2.0 Gr composite under current-carrying sliding conditions, where electrical current introduces additional degradation mechanisms. Therefore, due to its superior tribological performance, the Cu–HEA–2.0 Gr composite was selected for the present study, while the graphene-free Cu–HEA was employed as the reference material to directly assess the effect of graphene addition under current-carrying sliding conditions. It should be emphasized that the Cu–HEA–2.0 Gr composition is considered here as a model composition selected for investigating the effect of current-carrying sliding conditions, rather than as a finally optimized composition for electrical contact applications. Accordingly, the present study aims to evaluate whether the tribological advantages previously observed under conventional dry-sliding conditions can be maintained under current-carrying sliding conditions and to investigate the possible degradation mechanisms associated with friction and wear in the presence of electrical current.

## 2. Materials and Methods

### 2.1. Materials and Composite Fabrication Process

**Figure 1:**
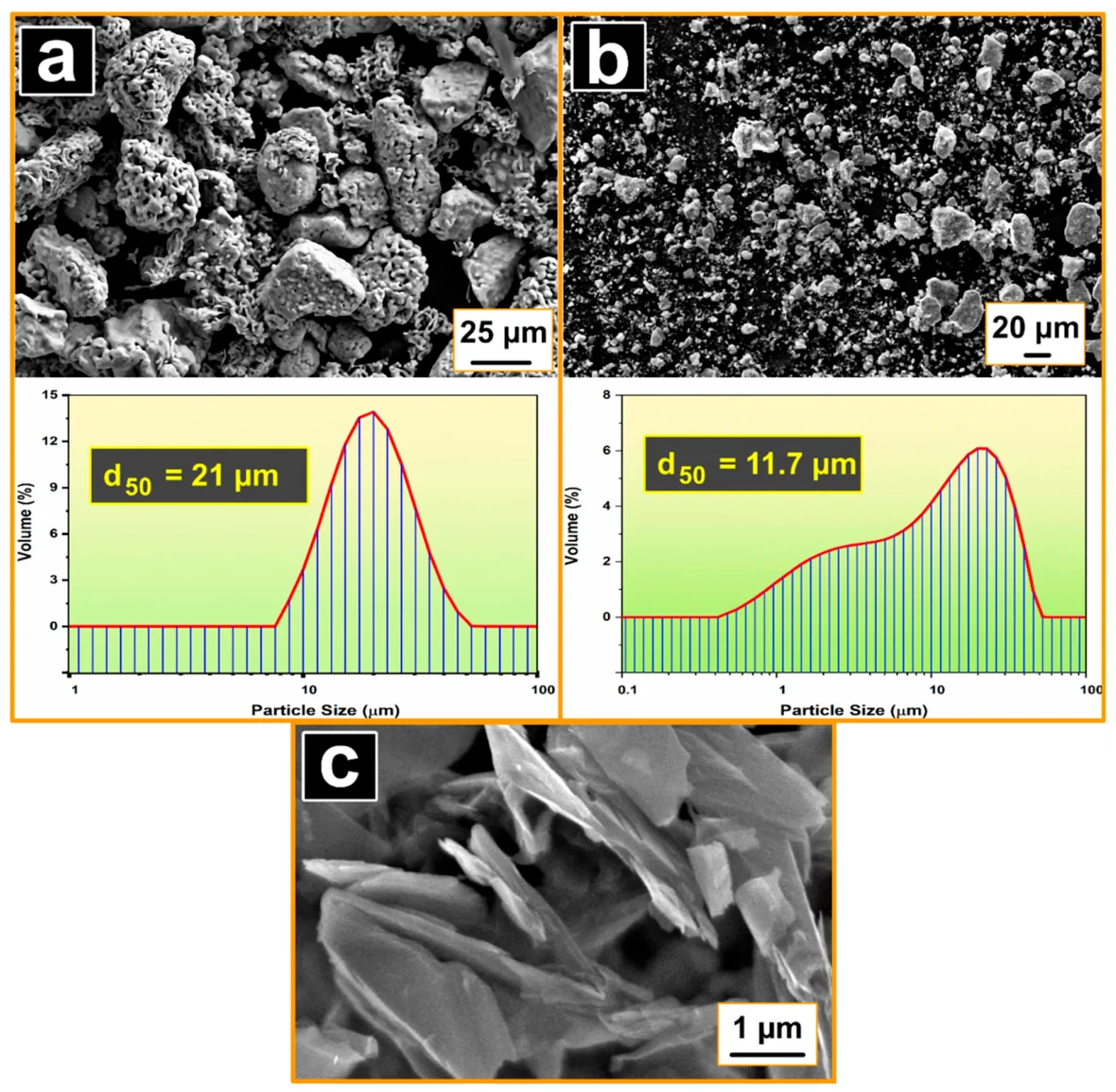
SEM images and particle size distributions of (**a**) as-received Cu powder, (**b**) 25 h milled AlCrFeCuNi HEA powder, and (**c**) as-received graphene powder.

In the present study, high-purity copper powder (99.99%, d50 = 21 µm, Nanografi, Ankara, Türkiye) was selected as the matrix phase. Fe–Ni–Al–Cu–Cr HEA powder with equal-mass composition (d50 = 11.7 µm) and graphene nanoparticles (d50 = 100 nm, Nanografi, Ankara, Türkiye) were incorporated as reinforcements. The synthesis of the HEA powder and the justification for choosing the 25 h milled condition were reported in our earlier work [20]. In our previous study, the influence of graphene content on the microstructural, mechanical, and dry-sliding tribological properties of Cu–HEA composites was systematically investigated, and the graphene-containing composition exhibiting the best overall tribological performance was identified [31]. Therefore, the present study specifically focuses on the graphene-free Cu–HEA and the optimized Cu–HEA–2.0 Gr composite in order to evaluate their behavior under current-carrying sliding conditions. The corresponding sample codes and reinforcement contents are summarized in Table 1. SEM observations and particle size analyses (Figure 1) indicate that the Cu powder has a highly homogeneous spherical morphology and narrow size distribution (Figure 1a), whereas the 25 h milled HEA powder possesses irregularly shaped particles and a wide bimodal particle size range (Figure 1b). The graphene particles, on the other hand, exhibit a layered nanosheet morphology (Figure 1c).

A planetary ball milling route was applied to obtain a homogeneous powder blend. For this purpose, the powder mixtures were processed in a Retsch PM100 mill (Verder company, Düsseldorf, Germany) at 400 rpm for 2 h with a ball-to-powder mass ratio of 10:1. Methanol (2 wt.%) was used as a process control agent to limit sticking and cold welding of the powders during milling. The selected milling parameters were determined from preliminary trials, where insufficient energy led to poor dispersion, while excessive milling severity caused pronounced cold welding. The milled hybrid powders were then compacted by spark plasma sintering in a Çelmak SPS–160 V unit (Çelmak Makina, Elazığ, Türkiye). Consolidation was carried out at 800 °C under 35 MPa for 4 min, employing a heating rate of 150 °C/min and a pulse on/off cycle of 12:2 in a vacuum and nitrogen regulated environment. These conditions were chosen after preliminary optimization to promote high densification, restrain grain growth, and reduce unfavorable interfacial reactions between Cu and the reinforcement phases.

### 2.2. Characterization

Following SPS consolidation, the hybrid composite specimens were subjected to standard metallographic preparation to enable reliable microstructural and phase characterization. Detailed examination of the bulk microstructure was performed using a Thermo Scientific Apreo 2S field-emission scanning electron microscope (FE-SEM) equipped with a Thermo Scientific UltraDry energy-dispersive spectroscopy (EDS) system (Thermo Scientific, Waltham, MA, USA). In addition, the worn surfaces and wear tracks formed after tribological testing were analyzed by SEM to elucidate the dominant wear mechanisms and the extent of surface deformation. The density of the Cu-based composite samples was determined according to Archimedes’ principle. Hardness measurements were carried out using the Brinell method (Innovatest Nemesis 9000, Maastricht, The Netherlands) under a load of 31.25 kg, and the reported values represent the average of six measurements taken from each sample. The electrical conductivity of the fabricated composites was evaluated with a portable SIGMASCOPE^®^ SMP10 (Fischer, Sindelfingen, Germany) conductivity meter. For each sample, three separate measurements were conducted and the average conductivity was reported as %IACS, based on the International Annealed Copper Standard.

### 2.3. Friction and Wear

**Figure 2:**
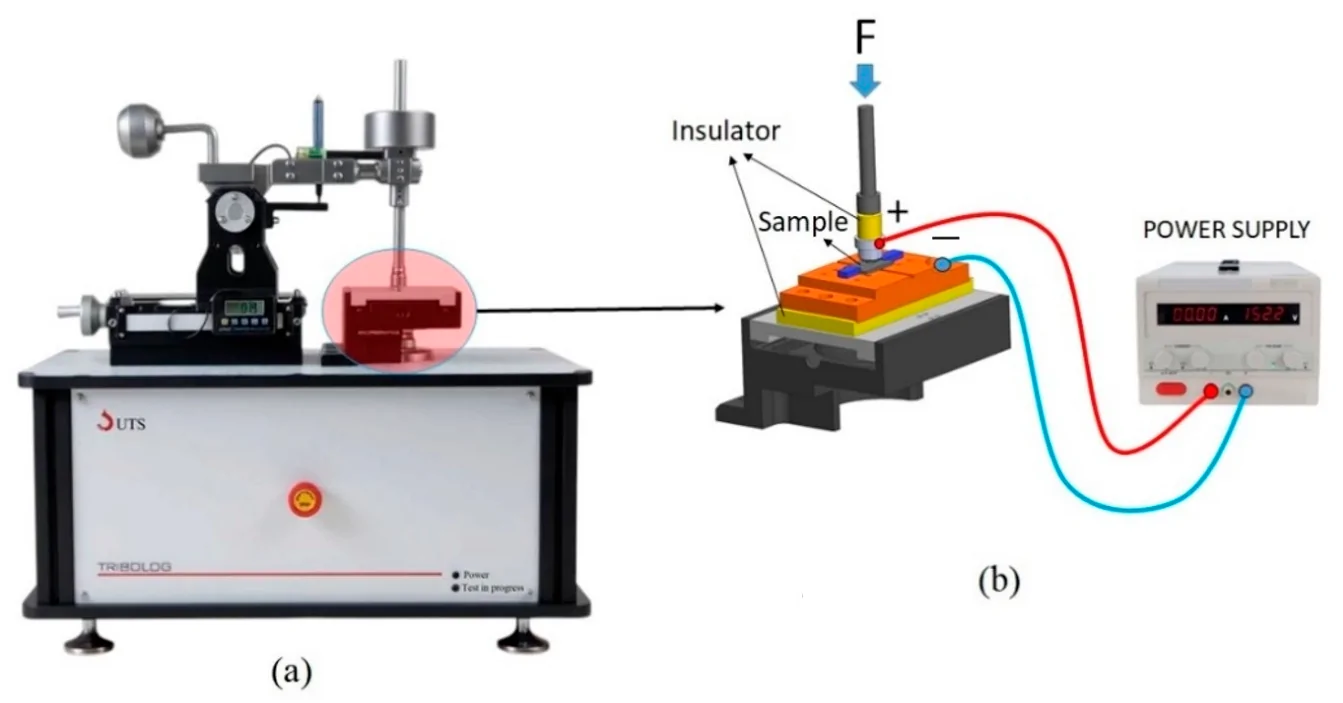
The experimental setup of the electrical sliding wear tests: (**a**) the UTS Tribolog multifunctional tribometer; (**b**) a schematic illustration of the ball-on-flat contact configuration with electrical current application.

Tribological tests were carried out in accordance with the ASTM G133 [*ASTM G133-22*; Test Method for Linearly Reciprocating Ball-on-Flat Sliding Wear. ASTM International: West Conshohocken, PA, USA, 2022] standard using a multifunctional tribometer (UTS Tribolog, Trabzon, Türkiye, Figure 2). A 100 Cr6 steel ball with a diameter of 6 mm was selected as the counterface material due to its high hardness (58–60 HRC), dimensional stability, and widespread use in tribological investigations of metallic contact materials. Its relatively stable mechanical behavior under repeated sliding and electrically assisted contact conditions enables reliable comparison of the tribological response of the developed Cu-based composites. The current levels of 0, 10, 30, and 50 A were selected to provide a progressive increase in electrical loading within the operating range of the experimental setup. Increasing current intensity is expected to promote Joule heating and may increase the likelihood of localized electrical discharge events and associated tribo-electrical surface degradation. All experiments were performed under a normal load of 10 N, a stroke length of 11 mm, a sliding frequency of 1 Hz, and a total of 5000 cycles. At least three repetitions were conducted for each condition to ensure reliability. All reported wear rate, average coefficient of friction, and electrical conductivity values are presented as the mean ± standard deviation of three independent measurements. Prior to testing, all samples were ground using 800–2500 grit SiC papers to obtain a uniform surface condition, while after testing, the specimens were cleaned in an ultrasonic bath to remove loosely adhered debris. The wear volume losses were determined by scanning the wear tracks using a Nanofocus µScan optical profilometric system, allowing both volumetric wear loss and 2D surface topographies to be obtained. The worn surfaces, wear debris, and counterball surface were comprehensively analyzed using field emission scanning electron microscopy (FESEM), and energy-dispersive X-ray spectroscopy (EDS) was employed where necessary to identify the chemical composition of selected regions.

## 3. Results

### 3.1. Microstructural Analysis 

**Figure 3:**
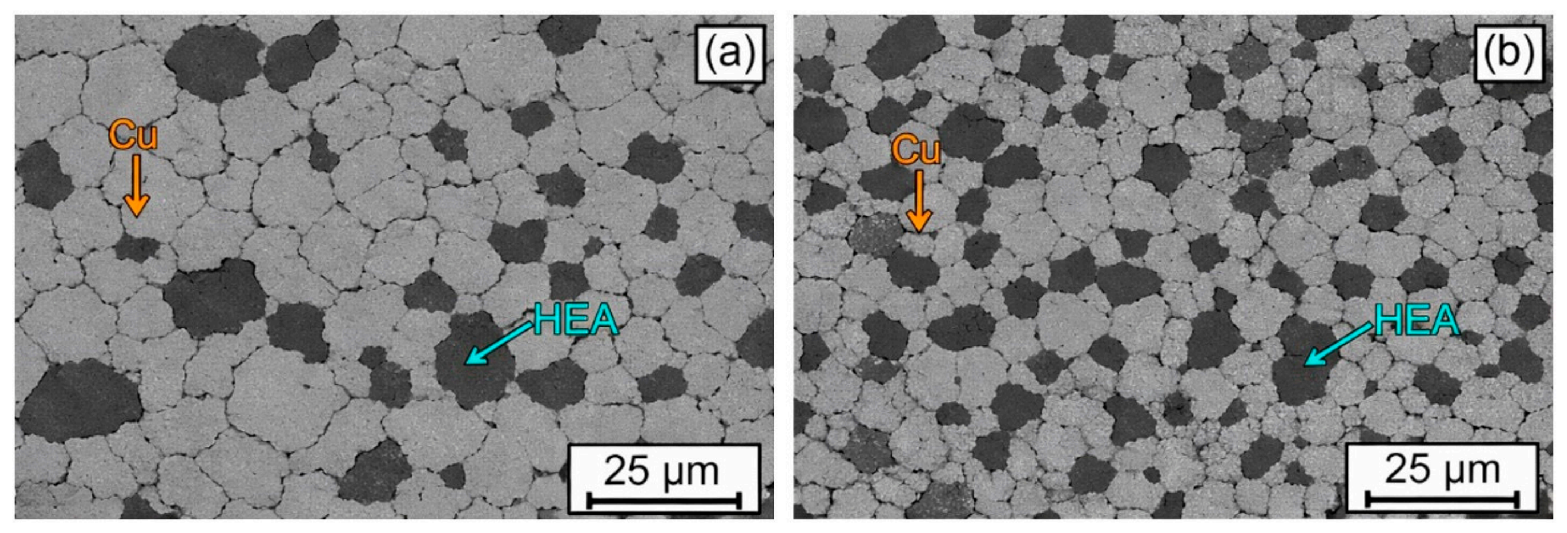
SEM images of the microstructure of Cu-HEA (**a**) and Cu-HEA-2.0 Gr (**b**) samples.

Microstructure SEM images of Cu-HEA and Cu-HEA-2.0 Gr composites are presented in Figure 3. Both samples produced via the SPS method generally exhibit a dense and homogeneous structure, with the reinforcement phases evenly distributed within the Cu matrix. In the Cu-HEA sample (Figure 3a), it is noteworthy that the AlCrFeCuNi HEA particles show a homogeneous distribution within the matrix and form a strong interfacial interaction with the matrix. The rapid heating and short holding time of the SPS process, which limits grain growth and ensures effective densification, plays a significant role in the formation of this ordered microstructure [32]. Furthermore, the irregular morphology of the HEA particles enhances mechanical interlocking, contributing to the load transfer mechanism. In the Cu-HEA-2.0 Gr sample (Figure 3b), the microstructure also exhibits a homogeneous distribution; however, a relatively finer-grained structure was obtained with the addition of graphene. The average grain size in the Cu-HEA sample was measured at approximately 13.70 µm, whereas the Cu-HEA-2.0 Gr sample exhibited an average grain size of approximately 7.27 µm [31]. Graphene is thought to limit grain growth by creating a pinning effect at grain boundaries due to its nano-sized structure and high specific surface area. This contributes to the formation of a finer and more refined microstructure. The literature also reports that graphene and similar carbon-based nanoreinforcements refine the microstructure by inhibiting grain boundary movement during sintering processes, thereby increasing mechanical strength [24]. This grain refinement, when evaluated within the context of the Hall-Petch mechanism, makes dislocation movement more difficult and consequently increases the material’s strength, albeit slightly. In this context, graphene addition contributes positively, albeit slightly, to the mechanical performance of the composite by maintaining microstructural homogeneity while simultaneously reducing grain size.

**Figure 4:**
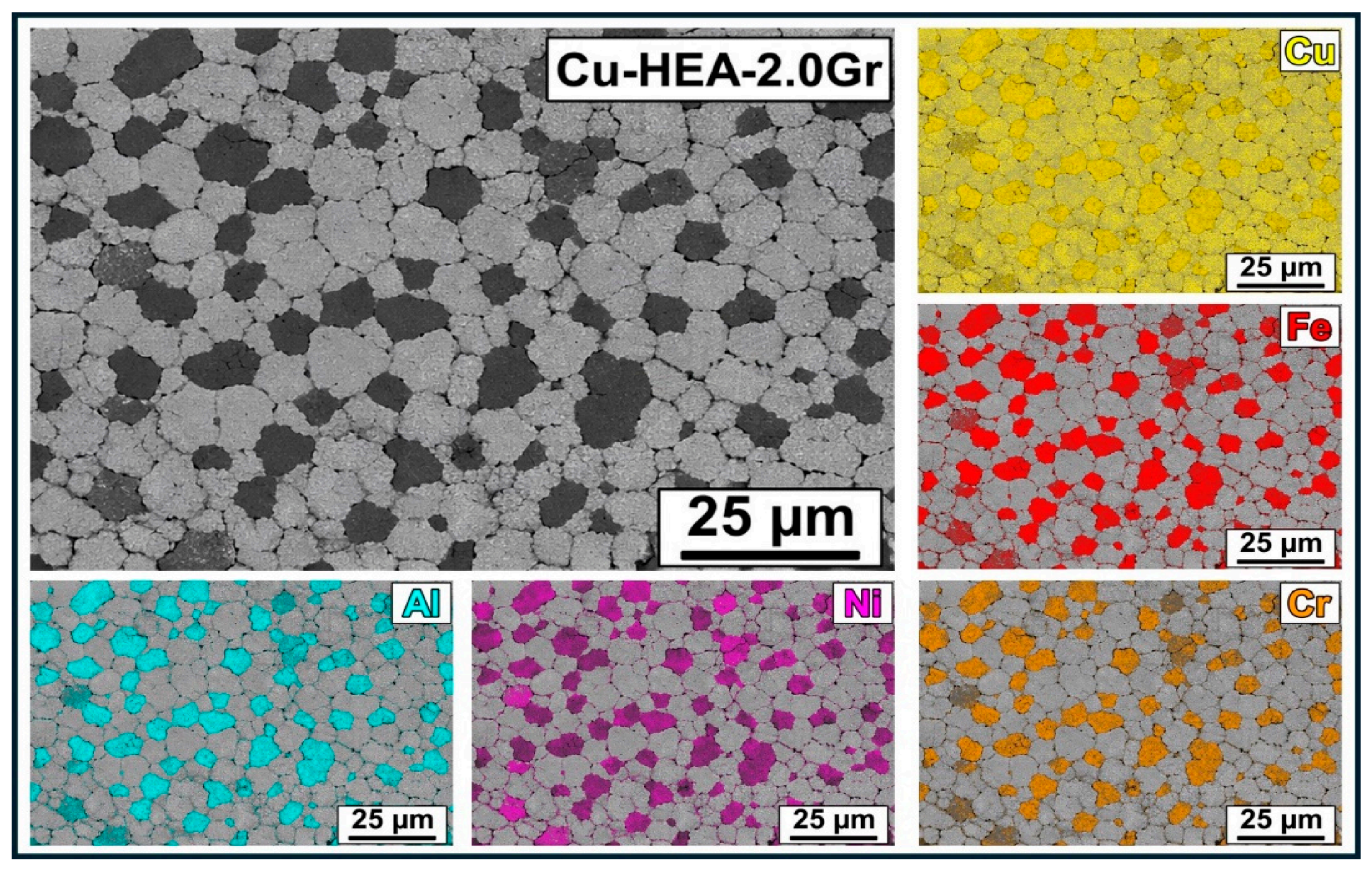
EDS analysis image of Cu-HEA-2.0 Gr sample.

The EDS analysis results given in Figure 4 confirm the distribution of HEA elements (Al, Cu, Cr, Fe, Ni) along with the Cu matrix in the Cu-HEA-2.0 Gr sample. However, direct detection of the graphene phase by EDS is limited. Due to the low atomic number and low X-ray emission efficiency of carbon-based phases, reliable determination in EDS analysis is quite difficult [33]. Therefore, the presence of graphene is mainly evaluated through morphological observations and indirect effects (e.g., increased porosity and possible formation of a tribological surface layer). EDS mapping results reveal that HEA particles are distributed within the matrix without showing significant segregation, which supports the effectiveness of the SPS process. From a microstructural perspective, the HEA phase acts as a hard and charge-bearing phase within the Cu matrix, while graphene is more of an interface phase that influences tribological behavior by forming slip planes. HEA particles increase hardness by limiting dislocation movement; however, this contribution of graphene is limited due to increased porosity. This explains why hardness values do not show a significant increase despite the addition of graphene. Similarly, the literature reports that when carbon-based reinforcements are used in high proportions, mechanical properties can be limited due to densification problems [34].

### 3.2. Density and Hardness Measurements

The experimental density, porosity, and hardness values of the produced samples are presented in Table 2. As can be seen, the Cu–HEA sample exhibited a porosity of 2.49% and a hardness of 127 HB, whereas the Cu–HEA–2.0 Gr sample showed a markedly higher porosity of 5.08% and a hardness of 129 HB. These results indicate that, although the addition of graphene significantly affected the consolidation response of the composite, it did not lead to any substantial improvement in hardness under the selected SPS conditions. In other words, while the graphene-containing sample maintained a hardness level comparable to that of its graphene-free counterpart, the increase in porosity clearly suggests that graphene posed a significant obstacle to full densification [35]. The relatively low porosity of the Cu–HEA sample confirms that SPS provided favorable conditions for the consolidation of the copper matrix combined with 20 wt.% AlCrFeCuNi HEA reinforcement. The simultaneous effects of rapid Joule heating, short holding time, and applied pressure are known to accelerate particle rearrangement and interfacial bonding while suppressing excessive grain coarsening [36,37]. In contrast, when 2 wt.% graphene was incorporated, the porosity increased to more than twice that of the Cu–HEA sample. It is well documented in the literature that a graphene content of 2 wt.% may represent a critical threshold in hybrid composites, beyond which the agglomeration tendency of graphene can adversely affect material properties. This behavior can be attributed to the intrinsic structural characteristics of graphene [34,38]. Owing to its nanosheet-like morphology, extremely high specific surface area, and strong tendency to agglomerate, graphene may accumulate in interparticle regions and act as a barrier between adjacent metallic particles. Such a barrier effect restricts direct Cu–Cu and Cu–HEA contact, suppresses local diffusion, and limits the formation of strong metallurgical bonds during sintering [39,40]. Moreover, the generally poor wettability between carbon-based reinforcements and metallic matrices may further reduce interfacial compatibility, thereby promoting pore retention rather than full densification. Although graphene is generally expected to enhance hardness by hindering dislocation motion and contributing to load transfer, the present results demonstrate that this strengthening effect remained highly limited [34]. Despite the incorporation of graphene, the hardness increased only marginally, from 127 HB to 129 HB, indicating that its potential reinforcing role was largely offset by the detrimental effect of increased porosity. Residual pores reduce the effective load-bearing area and act as local stress concentrators under indentation, thereby weakening the overall resistance of the material to plastic deformation [41,42].

### 3.3. Electrical Conductivity

**Figure 5:**
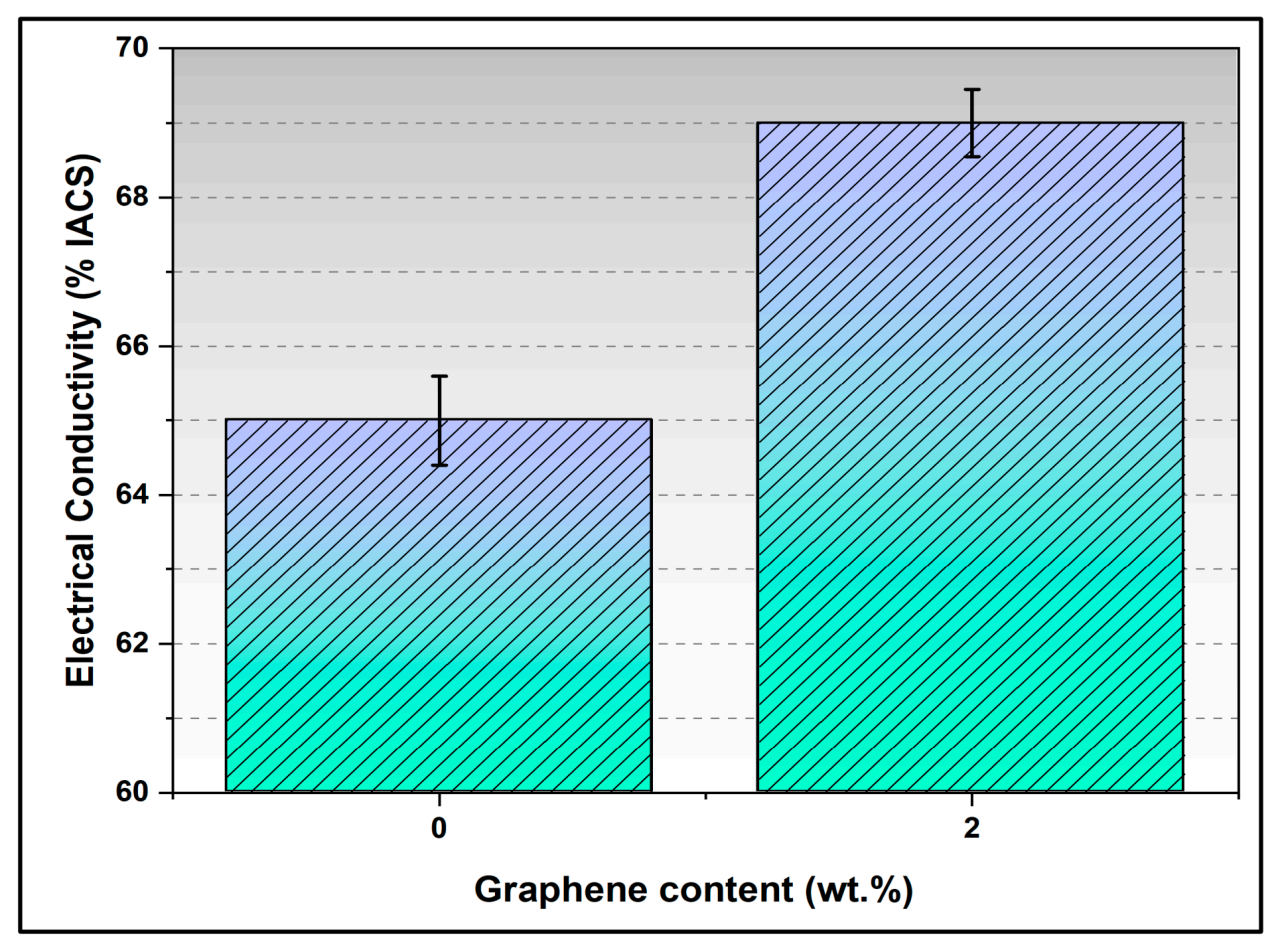
Comparison of the electrical conductivity (%IACS) values of the Cu-HEA and Cu-HEA-2.0 Gr composites as a function of graphene content.

The electrical conductivity values of the produced samples are presented in Figure 5. As clearly seen, the electrical conductivity increased slightly from 65% IACS for the Cu–HEA sample to 68% IACS for the Cu–HEA–2 Gr sample, corresponding to an improvement of approximately 4.6%. This result suggests that the incorporation of 2 wt.% graphene made a positive contribution to the electrical transport characteristics of the Cu-based hybrid composite. Given the intrinsically high electrical conductivity and carrier mobility of graphene, such an increase may be associated with its ability to promote local charge transfer and to provide supplementary conductive pathways within the matrix. However, the magnitude of this improvement remained limited, which can be explained mainly by the pronounced increase in residual porosity in the graphene-containing sample. In quantitative terms, the porosity rose from 2.49% to 5.08%, corresponding to an increase of nearly 104%. Such a substantial rise in pore content is highly significant for electrical transport, since pores act as non-conductive regions, reduce the effective cross-sectional area available for electron flow, and increase electron scattering throughout the consolidated structure. Therefore, although graphene inherently possesses superior electrical transport capability, the marked increase in porosity strongly restricted the extent to which this advantage could be reflected in the bulk electrical conductivity. In addition, the higher pore fraction likely disrupted the continuity of conductive pathways and weakened interparticle electrical contact within the SPS-consolidated microstructure. Under these conditions, the beneficial role of graphene in facilitating electron transport appears to have been largely offset by the adverse influence of incomplete densification. Accordingly, the relatively small increase from 65 to 68% IACS indicates that graphene did provide an electrically favorable contribution; nevertheless, this effect remained substantially suppressed by the more than twofold increase in porosity developed under the selected processing conditions.

### 3.4. Tribological Characterization

**Figure 6:**
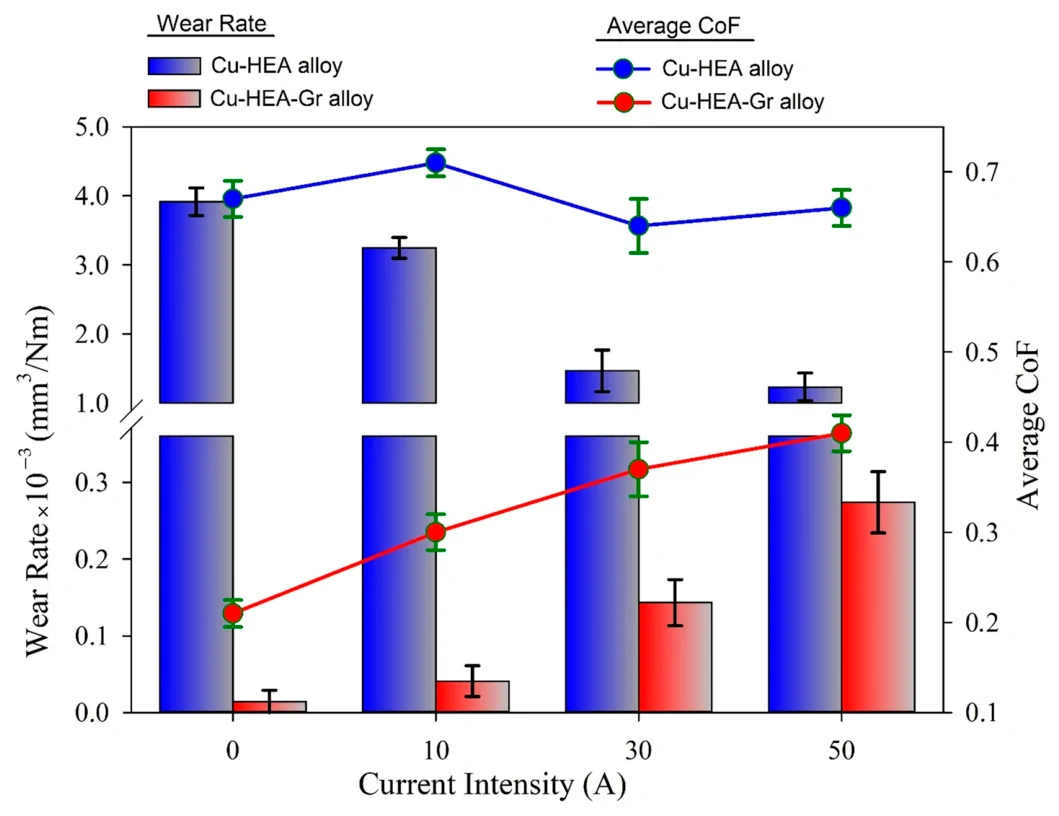
Comparison of average wear rate and average CoF for Cu-HEA and Cu-HEA-Gr composites as function of applied current intensity (0, 10, 30, and 50 A). Error bars represent ± one standard deviation obtained from three independent measurements (*n* = 3).

Figure 6 shows the combined evolution of wear rate and average coefficient of friction (CoF) for the Cu-HEA and Cu-HEA-Gr composites under increasing electrical current intensities from 0 to 50 A. Under purely mechanical sliding conditions (0 A), the Cu-HEA composite exhibits a wear rate of approximately 3.9 × 10^−3^ mm^3^/Nm, whereas the Cu-HEA-Gr composite performs significantly better wear performance with its wear rate dropping to a nearly negligible level of 0.02 × 10^−3^ mm^3^/Nm. This substantial reduction is consistent with the formation of an effective lubricating tribofilm that minimizes adhesive interaction between the 100 Cr6 steel counterface and the graphene-rich surface layer. The corresponding average CoF values further support this interpretation. Cu-HEA shows a relatively high CoF (0.68), while Cu-HEA-Gr exhibits a significantly reduced value (0.22). This decrease in the coefficient of friction supports the beneficial role of graphene in controlling interfacial shear under mechanical sliding.

As electrical current is gradually increased to 10 A, 30 A, and 50 A, the Cu-HEA and CU-HEA-2.0 Gr composites exhibit fundamentally different responses. For Cu-HEA, the wear rate decreases progressively from 3.9 × 10^−3^ mm^3^/N.m at 0 A to 3.2 × 10^−3^ mm^3^/N.m at 10 A, followed by a pronounced reduction to 1.4 × 10^−3^ mm^3^/N.m at 30 A and 1.2 × 10^−3^ mm^3^/N.m at 50 A, corresponding to an overall reduction of approximately 70% between 0 and 50 A. The apparent reduction in wear rate at higher current levels should not be interpreted as an improvement in the wear resistance of the Cu–HEA. Instead, increasing current intensity promotes severe Joule-heating-assisted thermal softening of the Cu-rich surface, leading to extensive plastic deformation and material redistribution within the wear track. Consequently, a considerable fraction of the deformed material remains on the worn surface or is displaced within the contact region rather than being completely detached as wear debris. As a result, the measured wear rate reflects a reduction in net volumetric material loss, even though the severity of surface deformation and damage continues to increase. The relatively stable CoF of Cu-HEA, fluctuating within 0.63–0.72 across all current levels, further supports this finding. Despite the substantial reduction in wear rate, the friction coefficient does not decrease proportionally. This indicates that the interfacial shear strength remains high, as the contact is dominated by plastically deformed metallic regions or discontinuous oxides rather than a continuous, low-friction oxide film.

However, unlike the Cu–HEA, the Cu–HEA–2.0 Gr composite exhibits a progressive increase in wear rate with increasing electrical current intensity. The wear rate increases from 0.02 × 10^−3^ mm^3^/N.m at 0 A to 0.04 × 10^−3^ mm^3^/N.m at 10 A, followed by a sharp increase to 0.14 × 10^−3^ mm^3^/N.m at 30 A and 0.27 × 10^−3^ mm^3^/N.m at 50 A, corresponding to more than a fifteen-fold increase compared to the initial condition. This behavior is likely associated with the gradual destabilization and degradation of the graphene-derived lubricating tribofilm under electrically assisted sliding conditions. Increased current promotes localized Joule heating and potential arc activity at asperity contacts, which can oxidize, disrupt, or partially remove the graphene-rich surface layer. As the protective tribolayer deteriorates with increasing current intensity, direct metal–metal contact between the Cu-based matrix and the 100 Cr6 counterface becomes more dominant, leading to increased interfacial shear stress and enhanced material removal at elevated current levels. Consistent with this behavior, the average CoF also increases from 0.22 to 0.42 over the same current range.

It should also be emphasized that the observed tribological behavior cannot be attributed solely to the presence of graphene. Graphene addition introduces two competing effects into the composite system. While graphene promotes the formation of a lubricating graphene-rich surface layer that reduces interfacial shear under low-current conditions, it also increases the porosity of the composite, thereby reducing the effective load-bearing area and local mechanical integrity, which may adversely influence wear resistance. Furthermore, although the increased porosity partially limits electrical transport, the Cu–HEA–2.0 Gr composite still exhibits a slightly higher electrical conductivity than the Cu–HEA. This improved electrical conductivity may contribute to a more stable electrical contact by reducing local current concentration and, consequently, the tendency for intermittent arc initiation. Therefore, the tribological response of the Cu–HEA–2.0 Gr composite should be interpreted as the combined consequence of the beneficial lubricating role of graphene together with the accompanying changes in densification and electrical transport characteristics, rather than as the isolated effect of graphene alone.

**Figure 7:**
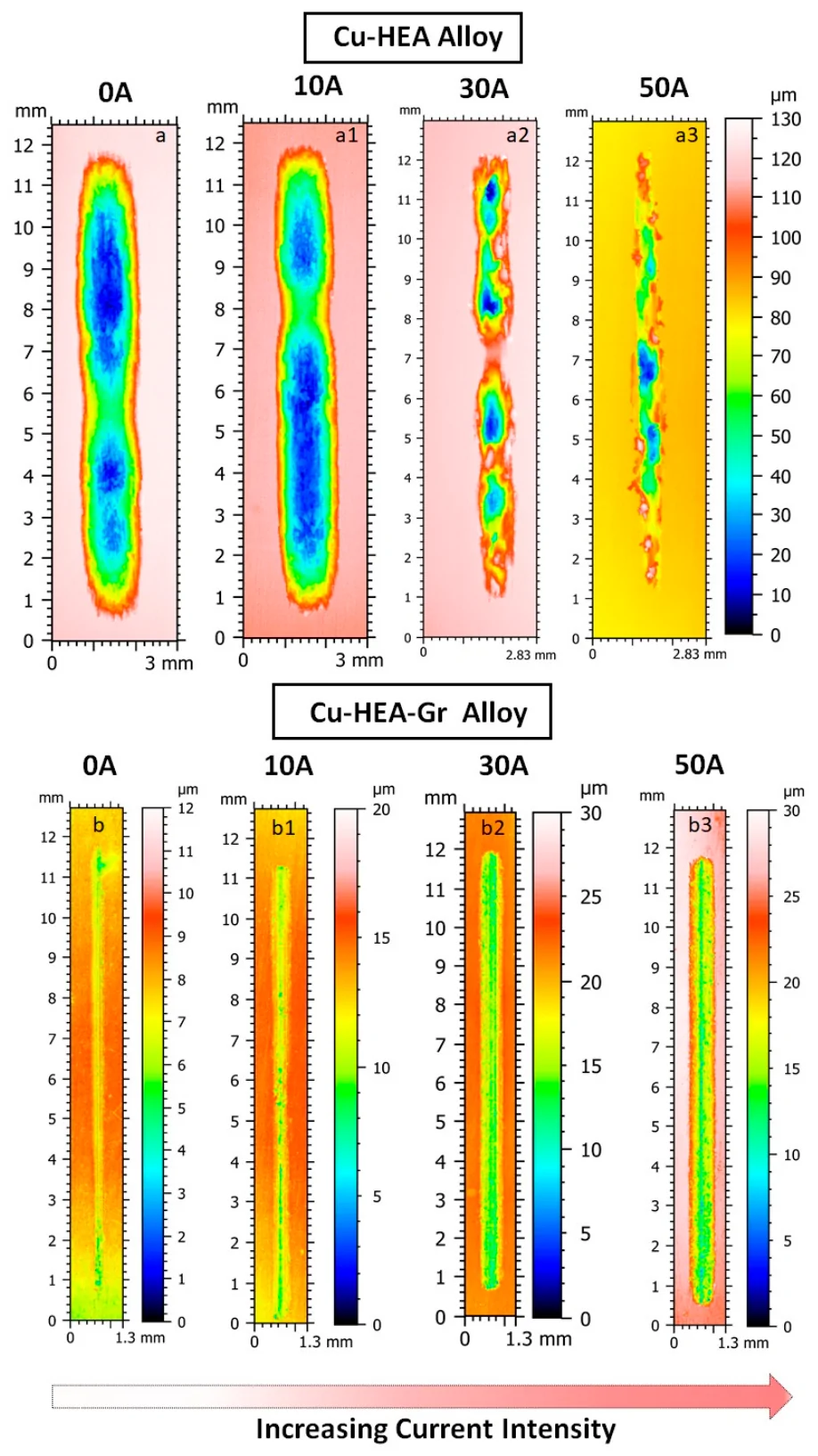
Two-dimensional surface profiles and topographic evolution of the wear tracks for the Cu-HEA intensities (**a**) 0A, (**a1**) 10A, (**a2**) 30A, and (**a3**) 50 A and Cu-HEA-Gr composites under increasing electrical current intensities (**b**) 0A, (**b1**) 10A, (**b2**) 30A, and (**b3**) 50 A.

The 2D surface profilometric views provide critical confirmation of the wear rate trends discussed previously and allow for direct visualization of the transition in degradation mechanisms with increasing electrical current. A clear correlation is observed between the measured wear rates and the evolution of wear track depth and width. For the Cu-HEA, the 0 A condition exhibits a wide and deep wear scar, consistent with the high wear rate (3.9 × 10^−3^ mm^3^/Nm). The track shows pronounced material removal and substantial depth variation across the contact length. At 10 A, although the wear track remains relatively wide, a slight reduction in maximum depth is noticeable, correlating with the moderate wear rate (3.2 × 10^−3^ mm^3^/Nm). A pronounced change occurs at 30 A and becomes even more apparent at 50 A. The overall depth and width of the wear track for 30 A and 50 A have decreased significantly compared to 0 A. The narrower and shallower topography directly validates the ~70% reduction in wear rate measured at 50 A (1.2 × 10^−3^ mm^3^/Nm). However, particularly at 50 A condition, the surface does not simply appear shallower; instead, distinct regions of material accumulation and smearing are clearly visible (Figure 7). These areas appear as elevated red zones relative to the reference surface level, indicating that the deposit height exceeds the nominal unworn surface. Such positive height deviations confirm that material has been plastically extruded and adhered onto the wear track rather than removed from it. The presence of these raised regions demonstrates that intense thermal softening occurred due to Joule heating at high current intensity, enabling uncontrolled plastic flow during sliding. Consequently, the apparent reduction in wear depth does not signify reduced damage severity, but rather reflects a transition from material removal to thermally assisted severe surface deformation and adhesive smearing on the contact surface.

In contrast, the Cu-HEA-Gr alloy exhibits considerably shallower wear tracks at 0 A, confirming the extremely low wear rate (0.02 × 10^−3^ mm^3^/N.m) (Figure 6). The wear scar is narrow and exhibits minimal depth variation, which is consistent with the presence of an effective proposed graphene-derived surface layer inferred from the combined SEM observations and tribological response under purely mechanical sliding. At 10 A, only a slight increase in track depth and width is visible, in agreement with the modest increase in wear rate (0.04 × 10^−3^ mm^3^/N.m). However, a clear transition is evident at 30 A and becomes more pronounced at 50 A. The wear track deepens and widens significantly compared to lower current levels, directly validating the sharp increase in wear rate (0.14 × 10^−3^ mm^3^/N.m at 30 A and 0.27 × 10^−3^ mm^3^/N.m at 50 A) (Figure 6). The enlarged wear scar observed at higher current intensities is consistent with the progressive disruption of the proposed graphene-rich surface layer, which may contribute to the increased volumetric material loss. It should be noted that the proposed tribofilm evolution is inferred from the wear morphology, SEM observations, and tribological response, since no direct chemical or spectroscopic characterization of the tribofilm was performed in the present study.

**Figure 8:**
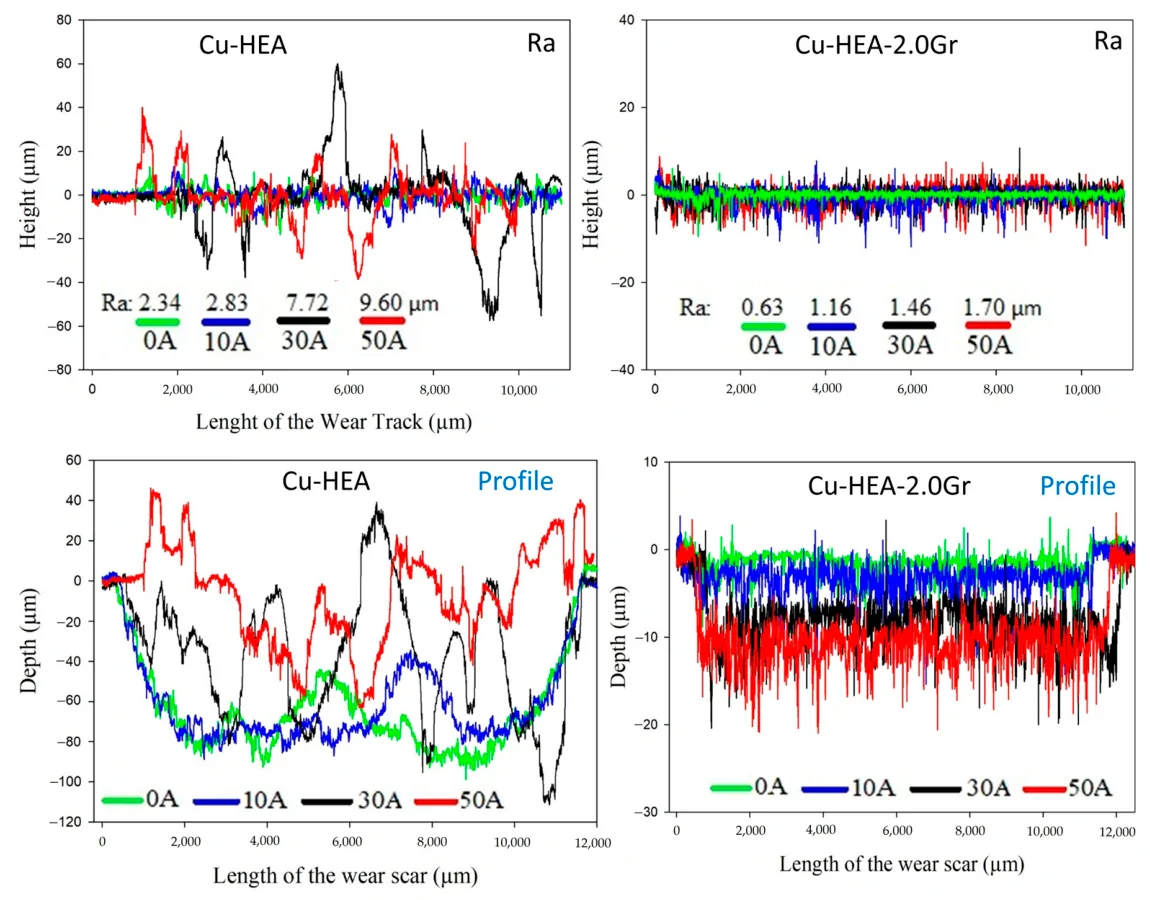
Comparison of surface roughness and longitudinal wear track profiles of Cu–HEA and Cu–HEA–Gr composites after sliding under different electrical current conditions.

Figure 8 presents the surface roughness (Ra) values and longitudinal wear track profiles extracted from the 3D surface topographies of the wear tracks shown in Figure 7. The Cu–HEA exhibited Ra values of 2.34, 2.83, 7.72, and 9.60 µm at 0, 10, 30, and 50 A, respectively. These results are in good agreement with the 3D surface topographies (Figure 7), which reveal increasingly heterogeneous wear track morphologies with increasing current intensity. In particular, the marked increase in Ra at 30 and 50 A is accompanied by pronounced height fluctuations and severe profile distortion along the sliding direction, indicating intensified surface deformation and non-uniform material redistribution under electrically assisted sliding conditions.

In contrast, the Cu–HEA–2.0 Gr composite exhibited considerably lower Ra values of 0.63, 1.16, 1.46, and 1.70 µm at 0, 10, 30, and 50 A, respectively. These lower roughness values are consistent with the relatively smooth and uniform 3D wear track morphologies observed in Figure 7, as well as the shallow longitudinal wear track profiles. The gradual increase in Ra and profile irregularity with increasing current suggests progressive deterioration of the sliding interface. This behavior is consistent with the gradual degradation of a proposed protective surface layer inferred from the tribological response, resulting in increased local height variations and a less homogeneous wear track morphology at elevated current intensities.

**Figure 9:**
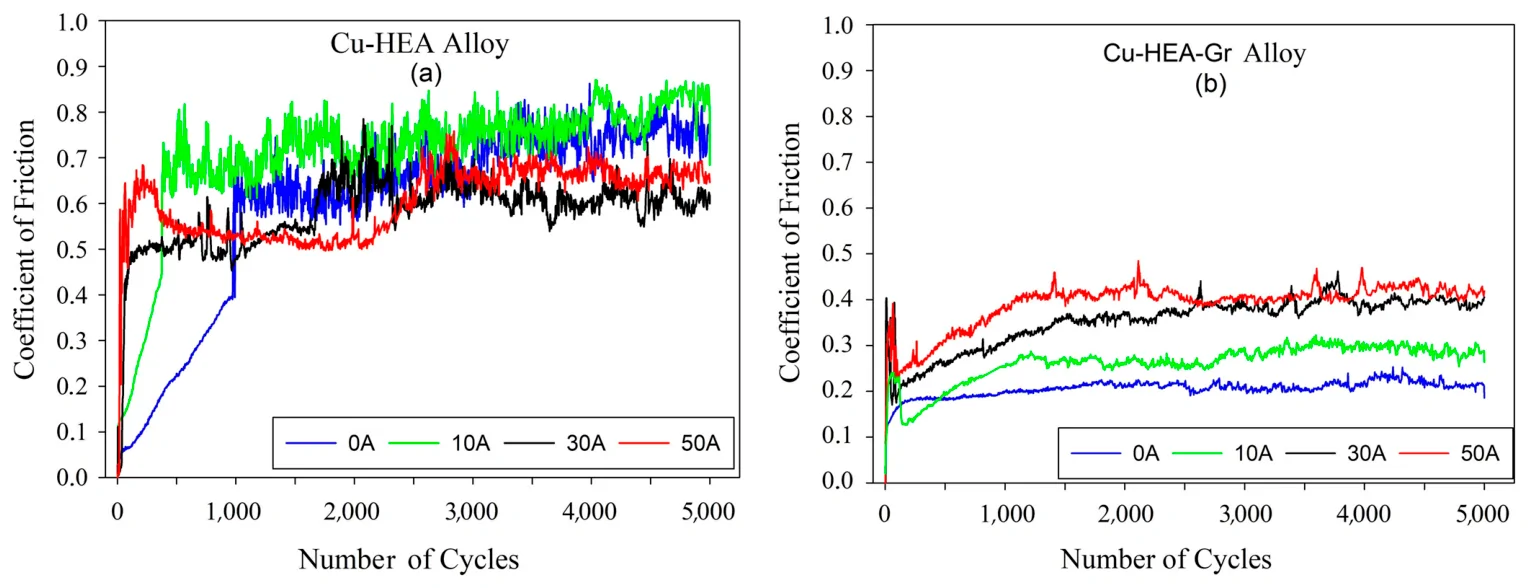
Variations in the coefficient of friction (COF) as a function of the number of sliding cycles for the (**a**) Cu-HEA and (**b**) Cu-HEA-Gr composites under different applied electrical currents.

The evolution of the coefficient of frictions (COF) as a function of sliding cycles are given in Figure 9. These graphs provide further insight into the current-dependent degradation mechanisms of the Cu-HEA and Cu-HEA-Gr composites. For the Cu-HEA, the friction curves exhibit a pronounced running-in stage followed by a relatively stable but fluctuating steady-state regime in all test conditions. At 0 A conditions, the COF gradually increases during the first 1000 cycles and then stabilizes in the range of 0.65–0.80. When electrical current is applied (10–50 A), the initial running-in stage is completed much more rapidly. The transition to the steady-state regime occurs significantly earlier depending on electrical current intensity. The friction curves of the Cu-HEA show that, regardless of the applied electrical current, the coefficient of friction exhibits a fluctuating behavior within the range of approximately 0.65–0.85 during the steady-state sliding stage (Figure 9a). Apart from differences observed during the initial running-in period depending on the testing conditions, no distinct change in the overall frictional character is evident. This indicates that the friction regime remains largely similar under all electrical loading conditions despite the variations observed in wear rate. This frictional behavior is also in good agreement with the wear track topographies and longitudinal profile analyses presented previously (Figure 8). As the applied current increased, the progressively rougher and more irregular wear track morphology of the Cu–HEA was accompanied by persistent fluctuations in the coefficient of friction, indicating an increasingly unstable sliding interface. Conversely, the Cu-HEA-Gr composite demonstrates a significantly different frictional character (Figure 9b). At 0 A, the COF rapidly stabilizes at a low level (0.20–0.25) with minimal fluctuations. This behavior is consistent with the presence of a stable graphene-based lubricating tribofilm between the contacting surfaces, as inferred from the combined tribological response and SEM observations. The smooth and steady friction behavior reflects efficient shear accommodation within the tribosystem. As the current increases to 10 A, the steady-state COF increases slightly to the range of 0.26–0.28; however, the frictional response remains stable. This behavior suggests that the proposed graphene-based tribofilm largely retains its lubricating function under this current intensity. This interpretation is further supported by the relatively low Ra values and smooth longitudinal wear track profiles observed under 0 and 10 A, which indicate a more homogeneous sliding interface. However, at high electrical current intensities (30 A and 50 A), the COF increases up to approximately 0.38–0.42 and shows more noticeable oscillations throughout the 5000 cycles of sliding. The simultaneous increase in the mean COF and the magnitude of frictional fluctuations is consistent with the progressive destabilization of the proposed lubricating tribofilm owing to the combined effects of Joule heating and possible arc activity. At higher current intensities, the gradual increase in surface roughness and profile irregularity (seen in Figure 8) is consistent with the increased fluctuations in the COF, suggesting progressive deterioration of the sliding interface. This increasingly unstable frictional response indicates that the contact conditions become progressively less uniform with increasing electrical current. Similar trends have been reported in the literature, where elevated thermal and electrical loads disrupt the integrity of proposed graphene-derived surface layers, leading to increased metal–metal contact and higher wear rates [43]. Unlike the Cu-HEA, the Cu-HEA-Gr composite shows a clear correlation between increasing average friction coefficient and increasing wear rate at elevated current intensities. This parallel trend supports the interpretation that graphene-rich surface layer destabilization under Joule heating and possible arc activity.

**Figure 10:**
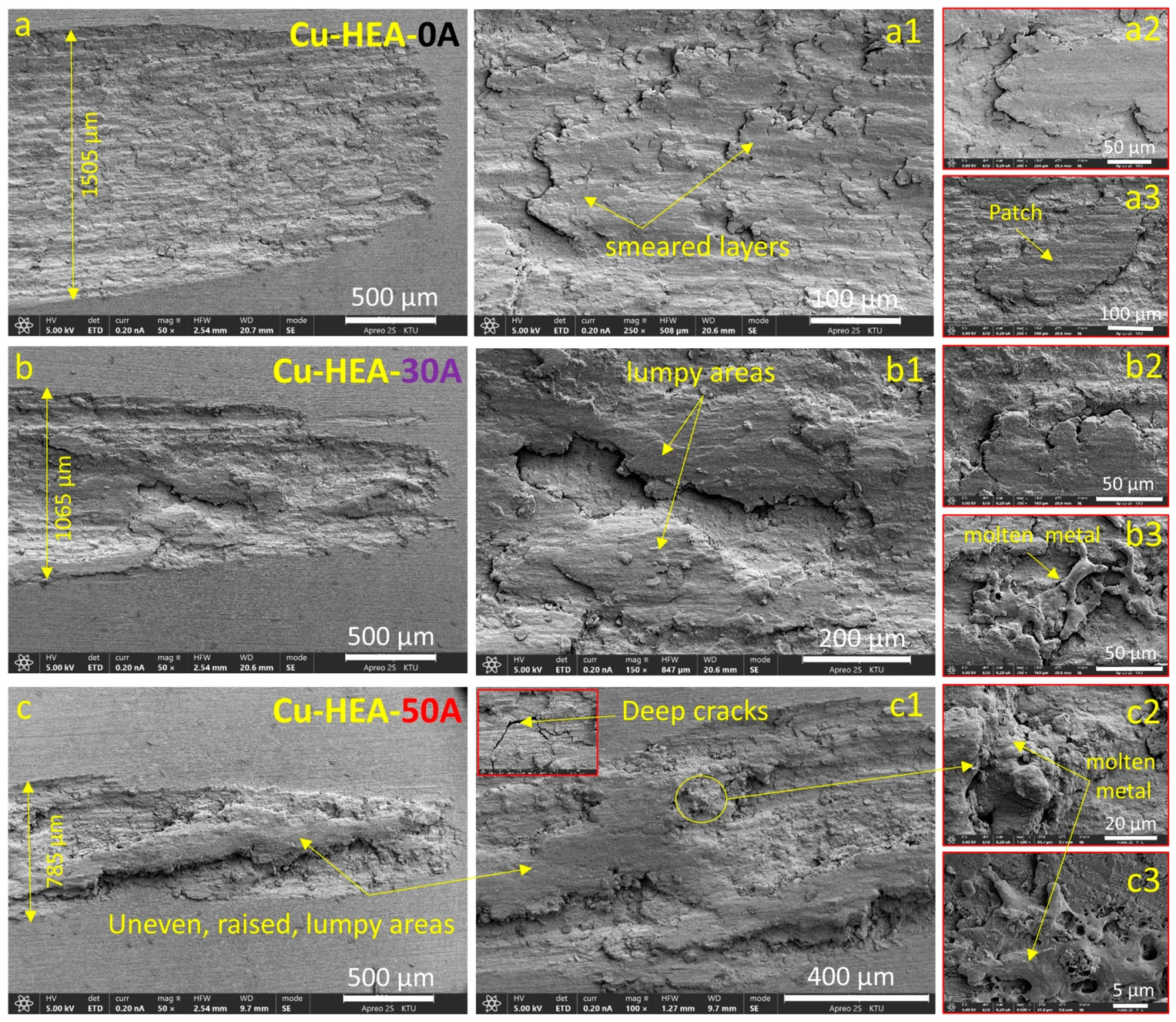
Representative SEM images of the worn surfaces of the Cu-HEA after electrical sliding tests conducted under different current intensities: (**a**,**a1**–**a3**) 0 A, (**b**,**b1**–**b3**) 30 A, and (**c**,**c1**–**c3**) 50 A.

Typical SEM micrographs of the worn surfaces of the Cu-HEA after electrical sliding tests at different current intensities are presented in Figure 10. The SEM observations provide important microstructural evidence explaining the trends observed in the wear rate, 2D surface topography and friction coefficient results. Under the 0 A condition, the worn surface exhibits pronounced plastic deformation characterized by extensive smearing and adhesive material transfer. As seen in Figure 10a,(a1–a3), the surface is covered with plastically deformed layers and patches of transferred material, indicating that adhesive wear dominates the degradation mechanism under only mechanical sliding. When the electrical current is increased to 30 A, the surface morphology changes significantly. Although the wear track width becomes narrower compared with the 0 A condition, the worn surface exhibits highly uneven, raised, and lumpy regions distributed along the sliding path (Figure 10b,(b1)). These features indicate that substantial thermal softening of the surface occurred due to deformation and electrical effects, leading to severe plastic deformation and extrusion of the softened metal. It is noteworthy that a substantial amount of the material does not detach from the tribological interface; instead, it accumulates unevenly across the wear track, forming distinct, raised, lumped areas. Consequently, much of the plastically deformed material remains within the contact zone as a smeared material rather than being removed from the system as debris. This interpretation is further supported by the longitudinal wear track profile and the surface roughness measurement (Figure 8), which reveal a shallower yet considerably more irregular wear track morphology, consistent with the accumulation of plastically displaced material within the contact zone rather than its removal as wear debris. This behavior explains why the volumetric wear loss appears to decrease despite the pronounced surface damage. The SEM observations are therefore consistent with the wear rate measurements and the 2D topographical images, which show a reduction in groove depth with increasing current intensity (Figure 6 and Figure 7). Furthermore, localized molten-like surface features are observed, particularly in Figure 10(b3). These morphological characteristics are consistent with localized arc-related thermal effects reported for electrically assisted sliding contacts. However, since no direct measurements of flash temperature or arc discharge activity were performed in the present study, this interpretation is based on the observed surface morphology together with previous research rather than direct experimental confirmation.

At the highest current level (50 A), the effects of thermal softening and arc degradation become even more pronounced. As shown in Figure 10c,(c1–c3), the worn surface displays extensive plastic flow accompanied by severe surface roughening, deep cracks, and widespread raised lumpy areas. These observations are also consistent with the increased surface roughness (Ra = 9.60 μm) and the highly irregular longitudinal wear track profile, indicating a progressively more heterogeneous contact interface under the 50 A condition (Figure 8). In addition to plastic deformation, clear evidence of localized melting is observed on the wear track (Figure 10(c2,c3)). The presence of resolidified molten-like regions and irregular melt features suggests that localized electro-thermal events may have occurred during sliding. The observed morphology may be associated with arc-related surface damage previously reported for electrically assisted sliding contacts, although direct confirmation of arc discharge activity is beyond the scope of the present work. These localized molten-like regions suggest that transient flash heating may have occurred at individual asperity contacts during electrical sliding [44]. As the current intensity increases, both Joule heating and arc heating contribute to significant thermal softening of the Cu matrix [45]. Furthermore, the severe deterioration of the surface morphology, together with the increased surface roughness and irregular wear track profile, is likely to promote increasingly unstable electrical contact conditions. Such conditions facilitate local current concentration and more frequent transient arc discharges, which further intensify localized heating and thermal softening of the Cu matrix. Previous studies have reported that transient flash temperatures associated with electrical discharge events may reach values on the order of 20,000 °C under specific electrical contact conditions [44]. However, such temperatures strongly depend on parameters including contact resistance, applied voltage, current density, contact geometry, and discharge duration. Therefore, the literature-reported value is cited here only to provide background information regarding the possible origin of the observed molten-like surface features and should not be interpreted as the actual temperature reached in the present experiments. The localized molten-like regions observed in Figure 10(c2,c3) are therefore interpreted as morphological features that may be associated with localized electro-thermal events occurring at asperity contacts during electrically assisted sliding.

**Figure 11:**
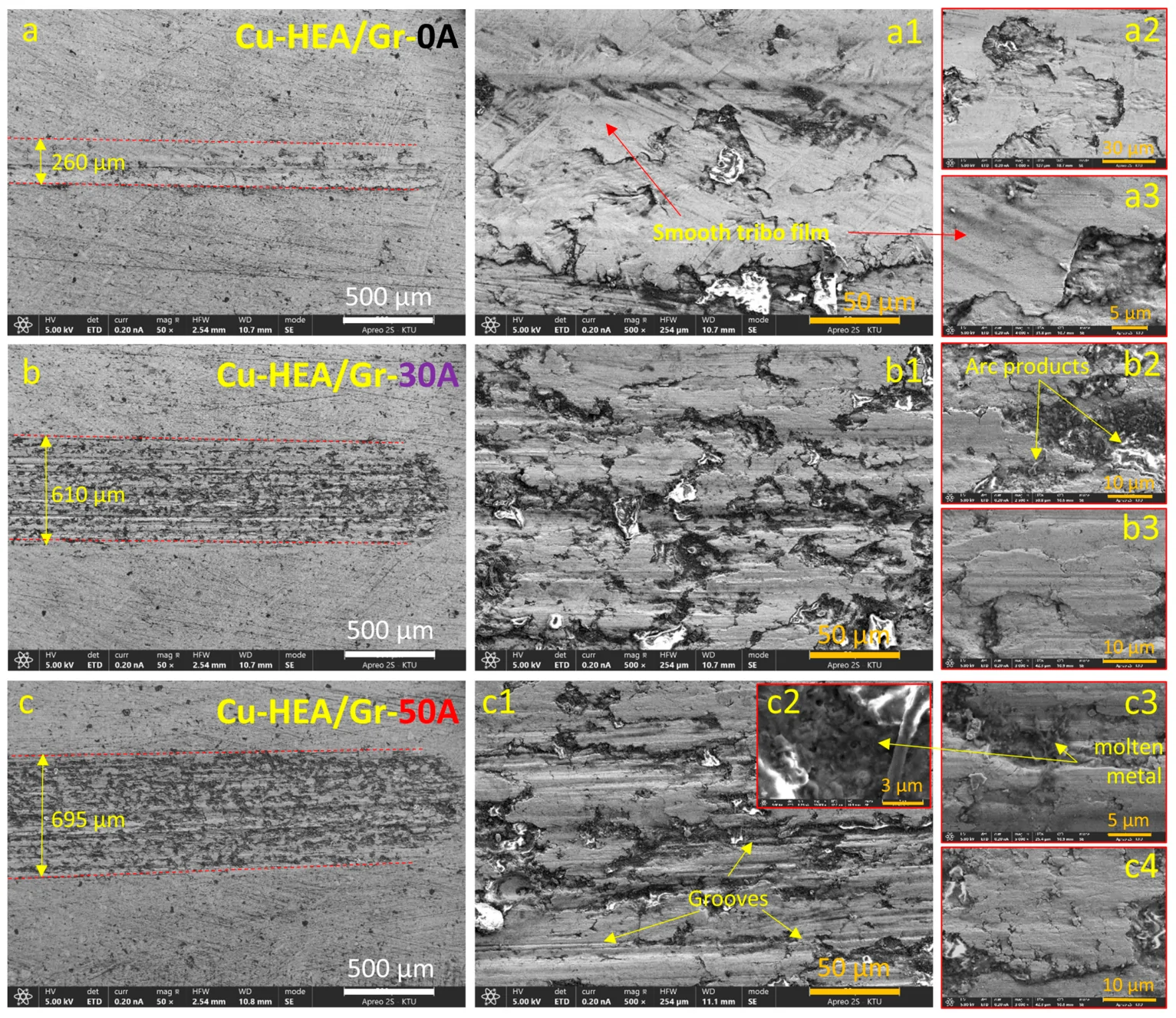
Representative SEM images of the worn surfaces of the Cu-HEA-Gr alloy after electrical sliding tests conducted under different current intensities: (**a**,**a1**–**a3**) 0 A, (**b**,**b1**–**b3**) 30 A, and (**c**,**c1**–**c4**) 50 A.

Representative SEM images of the worn surfaces of the Cu-HEA-Gr composite under different electrical current intensities are presented in Figure 11. Under the 0 A condition, the worn surface of the sample is largely covered by a smooth and continuous surface layer (Figure 11a,(a1–a3)), which is consistent with the smooth wear track morphology, low surface roughness (Ra = 0.63 μm), and shallow longitudinal profile presented in Figure 8. The widespread coverage of this surface layer suggests the presence of a graphene-rich transfer layer formed during sliding, which may reduce direct metal–metal contact between the mating surfaces. As a result, the friction coefficient remains very low (0.22–0.25) and highly stable throughout the sliding cycles (Figure 9b). Similar lubrication behavior has been widely reported for graphene-reinforced copper composites, where graphene sheets form a protective shear-accommodating tribolayer that stabilizes the friction response and reduces adhesive wear [43,46,47,48]. The relatively uniform distribution of this surface layer is also consistent with the low surface roughness, shallow wear track profile, and the stable COF evolution observed under purely mechanical sliding conditions. When the electrical current increases to the level of 30 A, noticeable changes begin to appear on the worn surface (Figure 11b,(b1–b3)). Although regions covered by the surface layer are still observed, local discontinuities and partial delamination of the film become evident (Figure 11b,(b1–b3)). These localized discontinuities suggest progressive deterioration of the sliding interface, which is accompanied by the increase in surface roughness (Ra = 1.46 μm) and the more irregular longitudinal wear track profile observed previously. However, the remaining surface-layer patches may still contribute to relatively stable sliding conditions between the contacting surfaces compared to the Cu-HEA. The partial coverage of the surface by the lubricating film also stabilizes the electrical contact, which likely suppresses frequent arc formation. This interpretation is further supported by the relatively low surface roughness (Ra = 1.46 μm) and the smoother longitudinal wear track profile compared with the Cu–HEA (Ra: 7.72 µm) tested under the same 30 A condition, indicating a more homogeneous contact interface during sliding. Consequently, clear arc erosion features are not prominently observed at 30 A. However, due to increased Joule heating, the overall wear damage becomes more pronounced than under 0 A conditions, which is reflected by the increased wear track width (~610 μm) and the higher wear rate measured at this current level (Figure 6).

When the applied current is further increased to 50 A, the stability of the proposed lubricating surface layer is substantially reduced, resulting in significantly more pronounced surface damage (Figure 11c,(c1–c4)). Although some localized surface-layer fragments remain, the worn surface becomes dominated by smeared metallic layers and plastically deformed regions. Compared with the 30 A condition, the wear track morphology becomes noticeably more heterogeneous, which is consistent with the increase in surface roughness (Ra = 1.70 μm) and the relatively irregular longitudinal wear track profile observed at 50 A. These changes suggest progressive deterioration of the contact interface with increasing electrical current. In particular, the formation of extensive smearing layers (Figure 11(c4)) suggests substantial thermal softening of the Cu-based matrix resulting from the combined effects of Joule heating and localized arc heating. The softened material is plastically displaced along the sliding direction, leading to the formation of smeared layers on the worn surface. This interpretation is further supported by the increased surface roughness (Ra = 1.70 μm) and the relatively irregular longitudinal wear track profile, both of which suggest a progressively less uniform electrical contact interface. Such conditions are likely to promote local current concentration, facilitating intermittent arc discharge and additional localized heating, thereby enhancing thermal softening, material redistribution, and the development of smeared surface layers. At the same time, arc-induced degradation becomes evident in the form of arc-related surface products and locally molten metal regions (Figure 11(c2,c3)). These micro-melted features are consistent with transient arc discharge events occurring at locations where electrical contact becomes increasingly intermittent under severe electrical loading. When electrical contact is locally interrupted, the current may bridge the contact gap through transient arc discharge, generating extremely high localized temperatures. The resulting features are consistent with localized melting and resolidification processes, as suggested by the irregular molten-like features observed on the worn surface. Furthermore, the higher friction coefficient together with the more pronounced fluctuations in the COF curves under the 50 A condition (Figure 9b) supports the interpretation of an increasingly unstable sliding interface, accompanied by more frequent metal-to-metal contact and intensified surface degradation.

In general, the present observations suggest that the graphene-containing surface layer plays an important role in improving the tribological behavior of the Cu–HEA composite under electrical sliding conditions. At low current intensities, the relatively smooth wear track morphology, low surface roughness, stable COF response, and the absence of severe surface damage are consistent with the presence of an effective lubricating surface layer. As the electrical current increases, the combined effects of Joule heating and possible localized electrical discharge events may progressively disrupt the continuity of the proposed surface layer, leading to increased surface roughness, plastic smearing of the thermally softened Cu matrix, and more pronounced surface deformation. This interpretation is in agreement with previous studies on graphene-reinforced copper-based composites under electrical sliding conditions, where progressive degradation of graphene-rich transfer layers has been associated with increasing current intensity [43,46].

**Figure 12:**
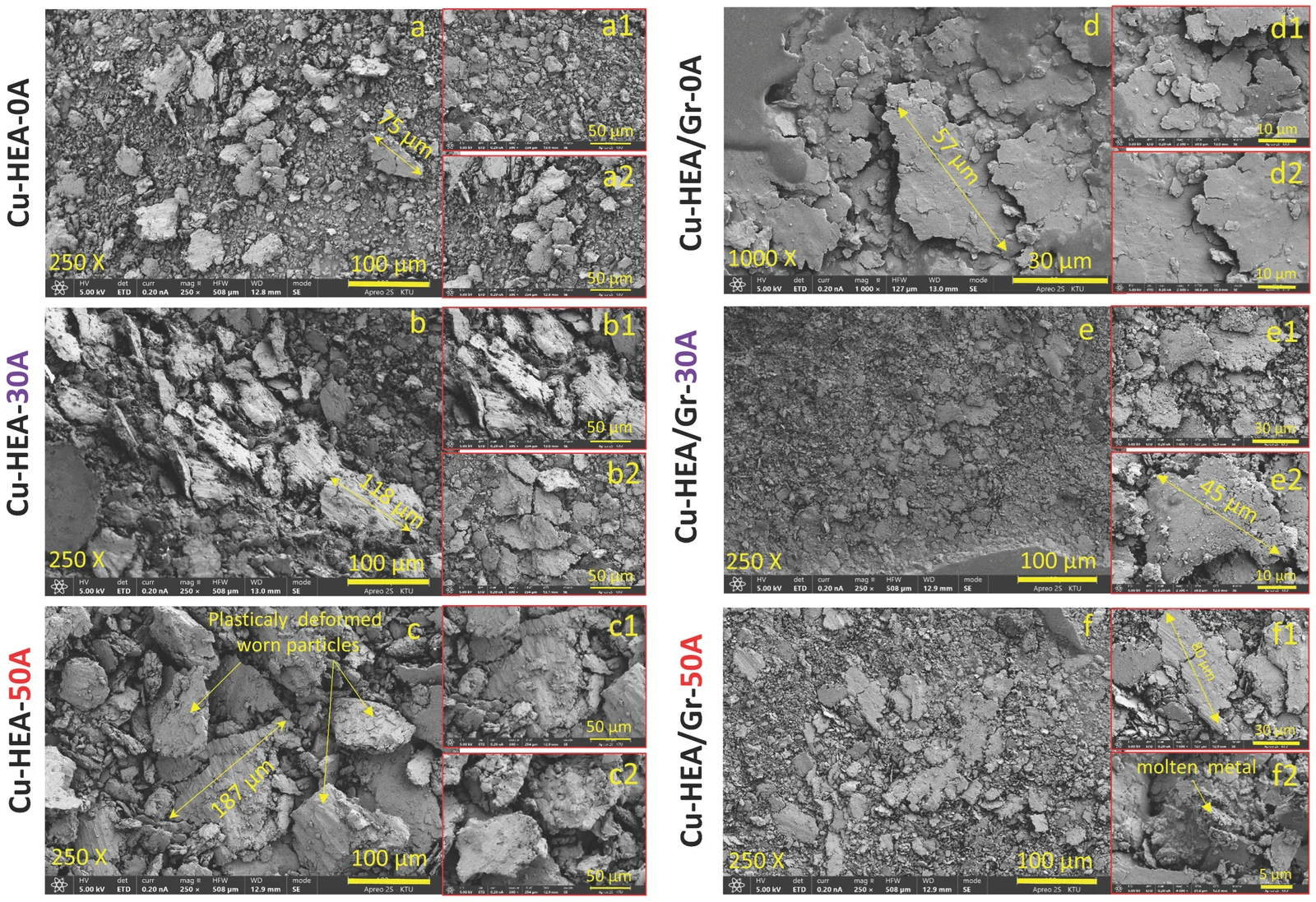
SEM images of wear debris generated from the Cu–HEA and Cu–HEA/Gr composites under different electrical current intensities: (**a**) 0 A; (**b**) 30 A and (**c**) 50 A for the Cu–HEA alloy, (**d**) 0 A; (**e**) 30 A; and (**f**) 50 A for the Cu–HEA/Gr alloy. The corresponding higher-magnification images are shown in (**a1**,**a2**), (**b1**,**b2**), (**c1**,**c2**), (**d1**,**d2**), (**e1**,**e2**), and (**f1**,**f2**), respectively.

Representative SEM images of the wear debris generated during sliding under different electrical current intensities are given in Figure 12. The debris morphology is dominated by relatively large and heavily plastically deformed particles for the Cu-HEA. Under the 0 A condition, the debris mainly consists of irregular, compact fragments formed by severe adhesive interaction and material transfer between the contacting surfaces (Figure 12a,(a1,a2)). As the electrical current increases to 30 A and 50 A, the debris becomes even coarser and more agglomerated (Figure 12b,(b1,b2),c,(c1,c2)). These large fragments may be associated with severe plastic deformation and the detachment of smeared surface layers. The presence of such large plastically deformed debris particles is consistent with the SEM observations of extensive surface smearing and material pile-up discussed in the previous sections. In contrast, significantly finer worn debris, typically appearing as thin flake-like or film-type particles, is produced in all test conditions for the Cu-HEA-Gr composite (Figure 12d,(d1,d2)). This morphology is consistent with the presence of a graphene-rich lubricating surface layer, which is likely to promote smoother sliding and limit the generation of large metallic fragments. However, as the electrical current increases, the debris size gradually increases, which is consistent with progressive disruption of the proposed graphene-rich surface layer together with the intensification of thermal effects (Figure 12e,(e1,e2)). Under the 50 A condition, noticeably larger fragments appear, which are likely generated by the fracture and detachment of smeared surface layers (Figure 12f,(f1,f2)). In addition, the morphology of some debris particles is also consistent with localized high-temperature surface events; however, these features cannot be conclusively attributed to arc erosion because electrical discharge activity and contact temperature were not directly monitored during the tests (Figure 12(f2)). These debris characteristics are consistent with the wear mechanisms discussed above and complement the observed surface morphologies, wear track topography, and wear rate measurements under high-current conditions.

**Figure 13:**
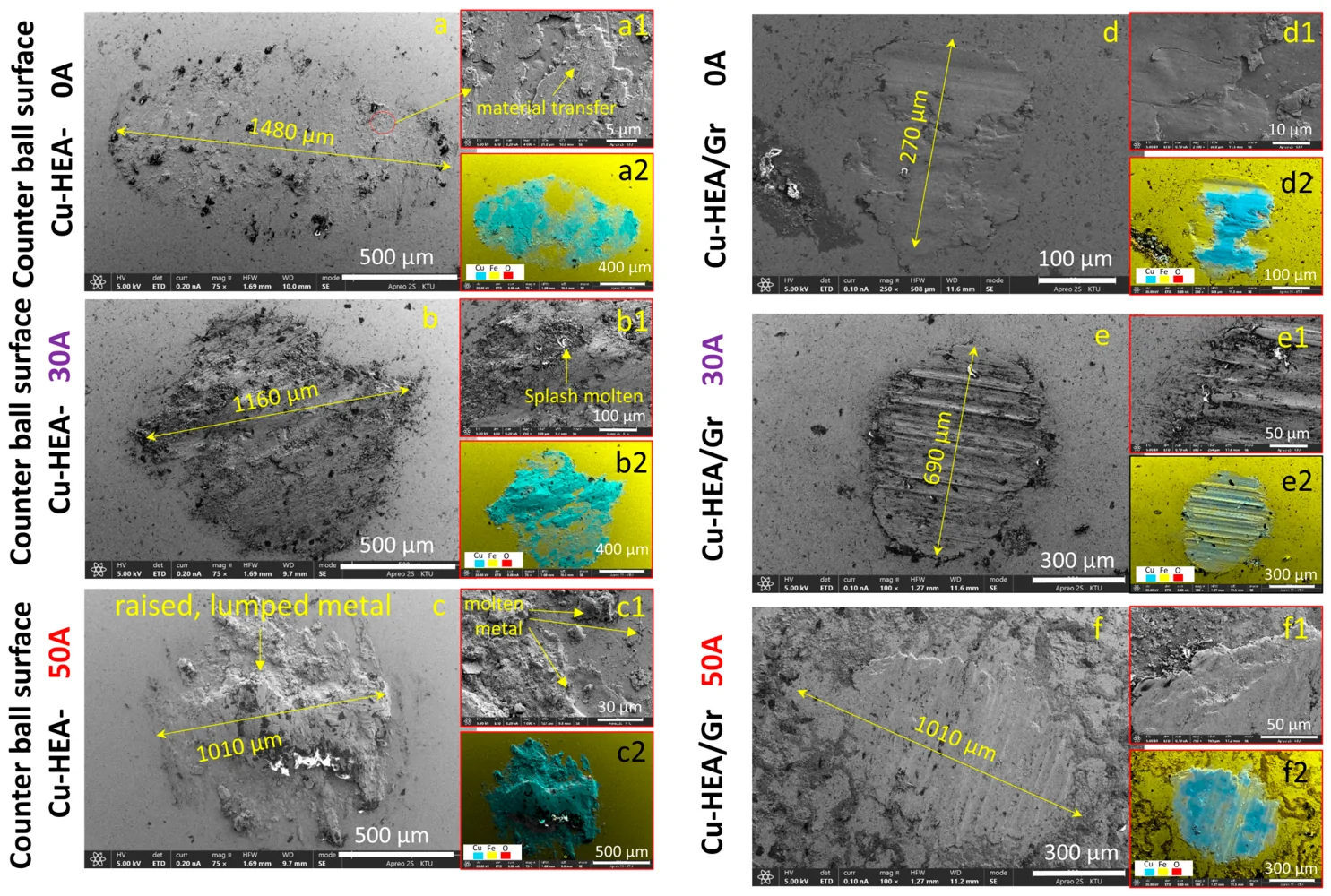
SEM images of the counter ball surfaces after sliding against Cu–HEA and Cu–HEA/Gr composites under different electrical current intensities: (**a**) 0 A; (**b**) 30 A and (**c**) 50 A for the Cu–HEA alloy, (**d**) 0 A; (**e**) 30 A; and (**f**) 50 A for the Cu–HEA/Gr alloy. The corresponding higher-magnification SEM images and optical images of the wear/transfer regions are shown in (**a1**,**a2**), (**b1**,**b2**), (**c1**,**c2**), (**d1**,**d2**), (**e1**,**e2**), and (**f1**,**f2**), respectively.

Representative SEM images of the counter ball surfaces after sliding under different electrical current intensities are presented in Figure 13. For the Cu-HEA, the counterface exhibits pronounced material transfer and severe smearing at the contact region. Under the 0 A condition, a clear transfer layer is already visible on the ball surface, and EDS analysis confirms that these adhered regions mainly consist of Cu-HEA material coming from the worn alloy surface (Figure 13a,(a1,a2)). The accumulation of transferred material becomes much more noticeable as the electrical current rises to 30 A and 50 A. Large adhered regions gradually built up and transformed into lumpy metallic deposits on the counterface (Figure 13b,(b1,b2),c,(c1,c2)). These surface features indicate intense adhesive effects combined with thermally induced softening of the metallic surface, which promotes extensive material transfer on the counter body. The formation of such irregular transfer layers can also explain the strong fluctuations observed in the friction coefficient, as these unstable surface features continuously modify the real contact area during sliding. On the other hand, the counterface surfaces obtained from the Cu-HEA-Gr composite exhibit relatively different. At 0 A, the ball surface is covered by a very thin and relatively uniform transfer layer, which is consistent with the presence of a graphene-rich surface layer transferred from the composite during sliding (Figure 13d,(d1,d2)). This transfer layer likely contributes to the low and stable friction behavior observed under purely mechanical sliding conditions (Figure 9b). With increasing electrical current (30 A and 50 A), the initially thin transfer layer becomes progressively disrupted and is accompanied by the formation of smeared metallic deposits as a result of combined electrical heating and tribological interaction (Figure 13e,(e1,e2),f,(f1,f2)). However, compared with the Cu-HEA, the extent of material accumulation and surface damage on the counterface remains significantly lower for the graphene-containing composite. This observation is consistent with the beneficial lubricating effect associated with the graphene-containing surface layer, which is likely to reduce severe adhesive material transfer and promote a more stable sliding interface even under electrically assisted conditions.

It should be noted that the proposed wear mechanisms associated with Joule heating and arc-related effects are inferred from the post-test microstructural, topographical, and tribological characterizations rather than being directly monitored during the sliding tests. Nevertheless, the consistency between the SEM observations, wear track profiles, surface roughness, wear rate measurements, and COF evolution provides strong support for these interpretations.

## 4. Conclusions

The main findings obtained from the microstructural, density, hardness, electrical conductivity and tribological evaluations carried out in this study are presented below:

1. The incorporation of 2.0 wt.% graphene effectively refined the Cu–HEA matrix by pinning grain boundaries, which suppressed grain growth and ensured a highly homogenous, fine-grained microstructure.
2. The incorporation of 2.0 wt.% graphene significantly influenced the physical and functional properties of the composites, increasing porosity from 2.49% to 5.08%, which limited the strengthening effect and resulted in only a limited hardness change from 127 HB to 129 HB.
3. For the Cu–HEA, increasing electrical current resulted in an apparent decrease in wear rate from 3.9 × 10^−3^ mm^3^/N·m at 0 A to 1.2 × 10^−3^ mm^3^/N·m at 50 A, although the coefficient of friction remained relatively high and fluctuating. This apparent decrease should not be interpreted as enhanced wear resistance. The increasing current promoted thermal softening of the Cu-rich matrix, facilitating severe plastic flow and redistribution of the material within the wear track instead of its complete removal as wear debris. Consequently, the measured volumetric material loss decreased even though the severity of surface deformation increased. This distinction is supported by the sharp increase in Ra from 2.34–2.83 μm at 0–10 A to 7.72–9.60 μm at 30–50 A, together with the pronounced height fluctuations and highly irregular longitudinal wear track profiles observed at the higher current levels.
4. Electrical current substantially modified the surface degradation behavior of the Cu–HEA. At higher current levels (≥30 A), thermally assisted plastic deformation and severe material redistribution became increasingly dominant, while some surface features were consistent with possible localized electrical discharge and high-temperature effects. These latter mechanisms are inferred from the observed morphology rather than directly measured during the tests.
5. The addition of graphene promoted the development of a graphene-rich lubricating surface layer, resulting in a low COF (0.22–0.25 at 0 A) and relatively stable friction behavior up to 10 A under electrical sliding conditions.
6. At higher current intensities (30–50 A), the beneficial lubricating effect of the graphene-rich surface layer gradually diminished, accompanied by increasing COF (0.38 Observed morphology–0.42) and more pronounced friction fluctuations for the Cu–HEA–2.0 Gr composite.
7. For the Cu–HEA–2.0 Gr composite, the wear rate increased progressively with increasing current, from 0.02 × 10^−3^ mm^3^/N·m at 0 A to 0.27 × 10^−3^ mm^3^/N·m at 50 A, accompanied by higher COF, increased surface roughness, and more pronounced surface deformation and features potentially associated with localized electro-thermal effects.

It should be noted that the findings of the present study are limited to the experimental conditions investigated, including a normal load of 10 N, a sliding frequency of 1 Hz, a 100 Cr6 steel counterbody, and an applied current range of 0–50 A. Therefore, additional investigations under different loading conditions, counterbody materials, sliding parameters, and application-specific operating conditions are required before extending these findings to practical electrical sliding contact systems, such as pantograph–catenary interfaces or other engineering electrical contacts.

## Figures and Tables

**Table 1:** The sample codes and milling parameters.

Sample Code	Milling Time (h)	Reinforcement Content		Milling Speed (rpm)	Ball-to- Powder Weight Ratio
		HEA (wt.%)	Graphene (wt.%)		
Cu-HEA	2	20	0	400	10:1
Cu-HEA-2.0 Gr	2	20	2.0	400	10:1

**Table 2:** The relative density and hardness values of samples.

Sample Code	Experimental Density (gr/cm^3^)	Porosity (%)	Hardness (HB)
Cu-HEA	7.88	2.49	127 ± 0.78
Cu-HEA-2.0 Gr	7.27	5.08	129 ± 1.12

## Data Availability

The original contributions presented in this study are included in the article. Further inquiries can be directed to the corresponding authors.
